# Antagonistic antimalarial properties of a methoxyamino chalcone derivative and 3-hydroxypyridinones in combination with dihydroartemisinin against *Plasmodium falciparum*

**DOI:** 10.7717/peerj.15187

**Published:** 2023-04-27

**Authors:** Tanyaluck Kampoun, Pimpisid Koonyosying, Jetsada Ruangsuriya, Parichat Prommana, Philip J. Shaw, Sumalee Kamchonwongpaisan, Hery Suwito, Ni Nyoman Tri Puspaningsih, Chairat Uthaipibull, Somdet Srichairatanakool

**Affiliations:** 1 Department of Biochemistry, Faculty of Medicine, Chiang Mai University, Chiang Mai, Thailand; 2 Medical Molecular Biotechnology Research Group, National Center for Genetic Engineering and Biotechnology (BIOTEC), National Science and Technology Development Agency, Pathum Thani, Thailand; 3 Department of Chemistry, Faculty of Science and Technology, Airlangga University, Surabaya, Indonesia; 4 Laboratory of Proteomics, University-CoE Research Center for Bio-Molecule Engineering, Universitas Airlangga, Surabaya, Indonesia; 5 Thailand Center of Excellence for Life Sciences (TCELS), Bangkok, Thailand

**Keywords:** Plasmodium, Antimalarial, Artemisinin, Chalcone, Hydroxypyridinone, Drug resistance, Ferredoxin

## Abstract

**Background:**

The spread of artemisinin (ART)-resistant *Plasmodium falciparum* threatens the control of malaria. Mutations in the propeller domains of *P. falciparum* Kelch13 (*k13*) are strongly associated with ART resistance. Ferredoxin (Fd), a component of the ferredoxin/NADP^+^ reductase (Fd/FNR) redox system, is essential for isoprenoid precursor synthesis in the plasmodial apicoplast, which is important for K13-dependent hemoglobin trafficking and ART activation. Therefore, Fd is an antimalarial drug target and *fd* mutations may modulate ART sensitivity. We hypothesized that loss of Fd/FNR function enhances the effect of *k13* mutation on ART resistance.

**Methods:**

In this study, methoxyamino chalcone (C3), an antimalarial compound that has been reported to inhibit the interaction of recombinant Fd and FNR proteins, was used as a chemical inhibitor of the Fd/FNR redox system. We investigated the inhibitory effects of dihydroartemisinin (DHA), C3, and iron chelators including deferiprone (DFP), 1-(*N*-acetyl-6-aminohexyl)-3-hydroxy-2-methylpyridin-4-one (CM1) and deferiprone-resveratrol hybrid (DFP-RVT) against wild-type (WT), *k13* mutant, *fd* mutant, and *k13 fd* double mutant *P. falciparum* parasites. Furthermore, we investigated the pharmacological interaction of C3 with DHA, in which the iron chelators were used as reference ART antagonists.

**Results:**

C3 showed antimalarial potency similar to that of the iron chelators. As expected, combining DHA with C3 or iron chelators exhibited a moderately antagonistic effect. No differences were observed among the mutant parasites with respect to their sensitivity to C3, iron chelators, or the interactions of these compounds with DHA.

**Discussion:**

The data suggest that inhibitors of the Fd/FNR redox system should be avoided as ART partner drugs in ART combination therapy for treating malaria.

## Introduction

**Figure 1:**
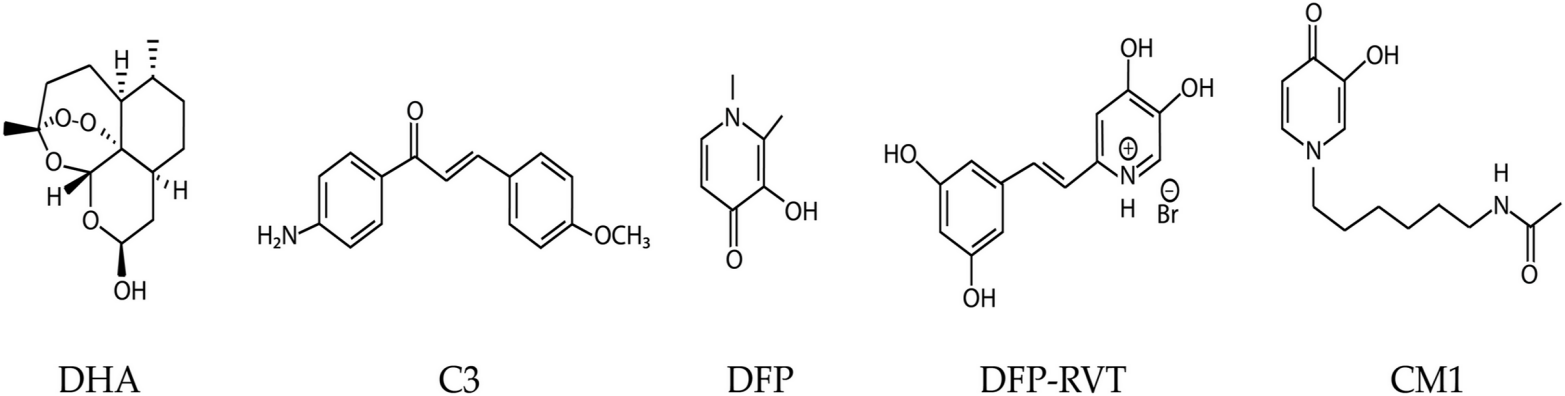
Chemical structures of dihydroartemisinin (DHA), compound 3 (C3), and 3-hydroxypyridinones; deferiprone (DFP), deferiprone-resveratrol (DFP-RVT), and 1-(*N*-acetyl-6-aminohexyl)-3-hydroxy-2-methylpyridin-4-one (CM1).

Artemisinin (ART)-based combination therapy is recommended by the World Health Organization for the first-line treatment of malaria in uncomplicated *Plasmodium falciparum* infections. ART and its derivatives are biotransformed by the liver to the active metabolite, dihydroartemisinin (DHA) (Fig. 1), which can be activated by iron, resulting in endoperoxide radicals that damage proteins *via* the formation of covalent adducts. The accumulation of damaged, polyubiquitinated proteins rapidly induces lethal endoplasmic reticulum stress (Bridgford et al., 2018). ART-resistant *P. falciparum* parasites emerged in Western Cambodia in the 2000s showing a slow clearance phenotype in malaria patients and an increased ring survival rate (Fairhurst & Dondorp, 2016; Tilley et al., 2016). Mutations in the kelch domain of the kelch13 (*k13*) gene have been established as genetic markers for ART-resistant malaria parasites (Ariey et al., 2014; Straimer et al., 2015); however, ART resistance is modulated by other genetic factors in some resistant parasites that do not contain *k13* mutations (Balikagala et al., 2017; Ikeda et al., 2018). A genome-wide association study identified variants in the ferredoxin (*fd*), apicoplast ribosomal protein S10 (*arps10*), multidrug resistance protein 2 (*mdr2*), and chloroquine resistance transporter (*crt*) genes as additional factors contributing to ART resistance, among which a missense *fd* mutation (*fd*-D193Y) was the most frequent variant among resistant parasites (Miotto et al., 2015).

Ferredoxin (Fd) is an iron-sulfur (Fe-S) electron carrier protein that functions with ferredoxin NADP^+^ reductase (FNR) in apicoplast metabolism, particularly isoprenoid biosynthesis, fatty acid desaturation, and heme oxygenation. However, only the isoprenoid biosynthesis pathway is essential during intraerythrocytic developmental stages (Gisselberg et al., 2013; Rohrich et al., 2005). The Fd-D97Y mutant protein (mutated residue corresponding to residue 193 in the full-length pre-processed Fd protein) has an increased binding affinity for FNR. This mutation reduces FNR function; hence, loss-of-function mutations in the Fd/FNR system may contribute to ART resistance (Kimata-Ariga & Morihisa, 2021). Isoprenoids are protein prenylation substrates, and prenylated proteins are required for vesicular transport to the digestive vacuole, including hemoglobin transport, in which K13 is thought to play an important role (Birnbaum et al., 2020; Mok et al., 2021). Hemoglobin degradation products are required for ART activation, and defects in hemoglobin trafficking cause ART resistance (Mukherjee et al., 2022). Therefore, loss of Fd/FNR function caused by *fd* mutation or chemical inhibition may enhance the effect of the *k13* mutation on the hemoglobin trafficking pathway, resulting in enhanced ART resistance. However, *fd/fnr* mutations are constrained, because this redox system is essential (Swift et al., 2022). Moreover, Fd/FNR is a validated antimalarial drug target (Qidwai et al., 2014; Suwito et al., 2014). The interaction between recombinant *Pf*Fd and *Pf*FNR proteins is inhibited *in vitro* by methoxyamino chalcone derivatives, of which the most potent compound, C3 (Fig. 1), exhibits 50% inhibition at 100 µM (Suwito et al., 2014).

In addition to Fd, iron is a cofactor for enzymes such as aconitase, oxidoreductases, and ribonucleotide reductase. Hemoglobin, myoglobin, and cytochromes contain an iron-prosthetic heme group. Iron also acts as a chemical catalyst for the generation of reactive oxygen species (ROS) *via* the Fenton and Haber–Weiss reactions. Iron chelators, such as desferrioxamine (DFO), 1,2-dimethyl-3-hydroxypyridin-4-one, deferiprone (DFP), and deferasirox (DFX) are clinically used for the treatment of patients with iron overload. Importantly, DFO, DFP, DFX, and other iron chelators, including 1-(*N*-acetyl-6-aminohexyl)-3-hydroxy-2-methylpyridin-4-one (CM1) and deferiprone-resveratrol hybrid (DFP-RVT) possess antimalarial activity by interfering with iron uptake, depleting the intraerythrocytic labile iron pool in parasites and expediting the phagocytosis of ingested parasites (Cabantchik, Moody-Haupt & Gordeuk, 1999; Chuljerm et al., 2021; Gordeuk, Thuma & Brittenham, 1994; Maneekesorn et al., 2021; Thipubon et al., 2015). Some antimalarial drugs such as quinine, mefloquine, and artesunate are antagonized by DFP (Pattanapanyasat et al., 2001).

In combination therapy, two different drugs are used to increase the efficacy of treatment, and the interaction between these drugs can be assessed by isobologram analysis (Huang et al., 2019). Drugs with a connection in their mechanisms of action, such as those acting on the same metabolic pathway, can exhibit synergistic or antagonistic interactions. Iron chelators are antagonistic to ART and related compounds (Antoine et al., 2014; Suwito et al., 2014), which can be explained by the iron-dependent activation of ART (Meshnick et al., 1993; Pattanapanyasat et al., 2001; Xie et al., 2016; Klonis, Creek & Tilley, 2013). Although the putative loss of function in the *fd*-D193Y mutant is associated with ART resistance, it does not affect the survival of *P. falciparum* parasites exposed to DHA (Kampoun et al., 2022; Stokes et al., 2021). Therefore, it is unclear whether the parasite genetic background, in particular, the allelic status of the *fd* and *k13* genes, affects the antimalarial potency of compounds targeting Fd/FNR and/or the interaction of these compounds with DHA. We hypothesized that the antimalarial potency of the chalcone derivative C3 and its interaction with DHA are affected by the genetic background of the parasite. Therefore, we evaluated the potencies of C3, DFP, DFP-RVT, and CM1, and the combinations of these compounds with DHA against *P. falciparum* wild-type (WT) and genome-edited parasites with *k13* and *fd* mutations. The iron chelators were used as reference ART antagonists for assessing the interaction of C3 with DHA. This study provides information regarding DHA interactions with compounds that target Fd/FNR.

## Materials and Methods

### Chemicals and reagents

D-Sorbitol, dimethylsulfoxide (DMSO), hydroxyethylpiperazine ethane sulfonic acid (HEPES), RPMI 1640 medium, and SYBR^®^ Green I were purchased from Sigma-Aldrich Chemical Company (St. Louis, MO, USA). Milli^®^-deionized water (DI) was purchased from Merck KGaA (Darmstadt, Germany).

### Drug and compounds

DHA (item No. 19846, MW = 284.4 g/mol) was purchased from Cayman Chemical Company (Ann Arbor, MI, USA). C3 compound (MW = 254.1183 g/mol) was synthesized by Dr. Hery Suwito as previously described (Suwito et al., 2014). DFP (MW = 139 g/mol) was purchased from Sigma-Aldrich (St Louis, MO, USA). DFP-RVT (MW = 340 g/mol) was kindly supplied by Dr. Yongmin Ma, School of Pharmaceutical and Chemical Engineering, Taizhou University, Taizhou, People’s Republic of China, which was synthesized by Xu and colleagues (Kampoun et al., 2022). 1-(N-Acetyl-6-aminohexyl)-3-hydroxy-2-methylpyridin-4-one or CM1 (MW = 266 g/mol), which is a DFP analog and an orally active bidentate iron chelator, was synthesized and kindly supplied by Dr. Kanjana Pangjit, College of Medicine and Public Health, Ubon Ratchathani University, Ubon Ratchathani, Thailand (Pangjit et al., 2015; Thipubon et al., 2015).

### Stock solutions of compounds

For the parasite growth inhibition assay, stock solutions of DHA, C3 compound, and DFP-RVT were prepared with 1% (*w/v*) DMSO as the solvent, whereas DFP and CM1 were dissolved in sterile DI water at 1,000 times the highest dose tested. Ten two-fold serial dilutions were prepared from the stock solutions. For the drug combination study, the drug or compounds were first dissolved in solvents at 2,000 times the highest dose tested. Five two-fold serial dilutions of the stock solutions were prepared. Stock solutions were sterilized using a sterile syringe filter (hydrophilic polyvinylidene difluoride membrane, 0.22 µm pore size, Sigma-Aldrich Chemicals Company, St. Louis, MO, USA) and stored at −20 °C.

### *Plasmodium falciparum* culture and synchronization

The 3D7 parasite line and the transgenic 3D7 parasite lines in this study, which were established in our earlier study, were cultured and maintained in the same conditions in which the parasites grew at the same rate in these culture conditions (Kampoun et al., 2022). Briefly, the parasites were cultured in complete RPMI 1640 medium pH 7.4 containing 2 mM L-glutamine, 25 mM HEPES, 2 g/L NaHCO_3_, 27.2 mg/L hypoxanthine, and 0.5% Albumax II using human O^+^ blood group erythrocytes (2–4% hematocrit). Parasite cultures were incubated in 90% N_2_, 5% CO_2_, and 5% O_2_ at 37 °C and synchronized to the ring stage using 5% D-sorbitol treatment. Parasite developmental stage and viability were routinely assessed by microscopic examination of Giemsa-stained thin smear films.

### *Plasmodium falciparum* growth inhibition assays

Before use, stock solutions of the compounds were 100× diluted in a complete RPMI 1640 medium. Ten microliters of each dilution were added to each well of a black 96-well plate. Synchronized ring-stage *P. falciparum* was cultured in complete RPMI 1640 medium at 1% parasitemia and 2% hematocrit, which was dispensed at 90 µL/well to perform the treatments. The parasite was treated in technical duplicates with various doses of the test compound in a black 96-well plate, while a mock treatment (parasite suspension in culture medium with 0.1% DMSO) was also performed. The mock treatment was assigned as 100% parasite growth. The treated parasites were incubated under standard culture conditions for 48 h and the parasite survival rate was determined using the malaria SYBR^®^ Green I-based fluorescence (MSF) assay as described previously (Maneekesorn et al., 2021).

For the MSF assay, 0.2 µL of SYBR^®^ Green I was diluted in 1 mL of lysis buffer solution containing 20 mM Tris-HCl, 5 mM EDTA, 0.008% (*w/v*) saponin, and 0.08% (*v/v*) Triton X100. SYBR^®^ Green I solution (100 µL) was added to each well of a 96-well black microplate (Corning^®^ Product number CLS 3601, polystyrene flat-bottom type, Merck KGaA, Darmstadt, Germany). The plate was mixed using a MixMate^®^ Eppendorf machine (Eppendorf SE, Hamburg, Germany) at 1,000 rpm for 30 s and incubated for 1 h in the dark at room temperature. Fluorescence intensity (FI) was measured using a fluorescence multi-well plate reader (Beckman Coulter AD340C; Beckman Coulter Inc., Brea, CA, USA) with excitation and emission wavelengths of 485 and 530 nm, respectively. The concentration of the test compound with 50% inhibition of growth (IC_50_) was calculated using the drc R package (Ritz & Streibig, 2005).

### Combination treatment against *P. falciparum* growth

Working solutions of the drug or compound were mixed in a 1:1 ratio (5 µL each) in a checkerboard manner in a black 96-well plate, in which the concentration of one drug or compound was fixed while the concentrations of the other were varied. The parasite suspension (1% rings, 2% hematocrit) was then added to each well (100 µL/well in total) and cultured for 48 h under standard conditions. The parasite growth was determined using the MSF assay as described above. Data were obtained from at least three independent replicates, with two technical replicates per experiment.

### Isobologram analysis of drug combination

The interaction of DHA with Fd-FNR inhibitor or iron chelator was assessed by isobologram analysis of IC_50_ values. The FIC indices were calculated from the ratio of the IC_50_ value obtained from the combination treatment to that obtained from the single compound treatment. The ΣFIC for the combination is the summation of an individual drug or compound. The FIC value was calculated as follows:

$$\matrix{
   {\Sigma {\rm{FIC}}} & { = [({\rm{I}}{{\rm{C}}_{{\rm{50}}}}\;{\rm{of}}\;{\rm{A}}\;{\rm{in}}\;{\rm{the}}\;{\rm{mixture}}) \div [{\rm{I}}{{\rm{C}}_{{\rm{50}}}}\;{\rm{of}}\;{\rm{A}}\;{\rm{alone}})]}  \cr 
   {} & { + [({\rm{I}}{{\rm{C}}_{{\rm{50}}}}\;{\rm{of}}\;{\rm{B}}\;{\rm{in}}\;{\rm{the}}\;{\rm{mixture}}) \div [{\rm{I}}{{\rm{C}}_{{\rm{50}}}}\;{\rm{of}}\;{\rm{B}}\;{\rm{alone}})].}  \cr 

 } $$

The FICs of the combinations from at least three individual experiments were used to calculate the mean values of ΣFIC. Interactions were assigned as synergistic (ΣFIC < 0.9), additive (0.9 < ΣFIC < 1.1), or antagonistic (ΣFIC > 1.1), with 1.1 < ΣFIC < 1.2 representing slight antagonism, 1.2 < ΣFIC < 1.45 representing moderate antagonism, 1.45 < ΣFIC < 3.3 representing antagonism, 3.3 < ΣFIC < 10 representing strong antagonism, and ΣFIC > 10 representing very strong antagonism (Chou, 2006; Crisafulli et al., 2021).

### Statistical analysis

Data for each experiment were obtained from at least three independent replicates. The IC_50_ values of the mutant parasites were compared with those of the parental 3D7 parasite using drc R package by pair-wise t-tests with Bonferroni–Holm *post-hoc* correction of the *P*-values, in which *P* < 0.05 was considered significant (Ritz & Streibig, 2005). The mean ΣFICs of *fd*-D193Y parasites were statistically compared with the mean ∑FIC of *fd* wild-type parasites using GraphPad Prism 8.3.0 software by unpaired t-test with Welch’s correction.

## Results

### Single compound sensitivity test of *P. falciparum*

**Figure 2:**
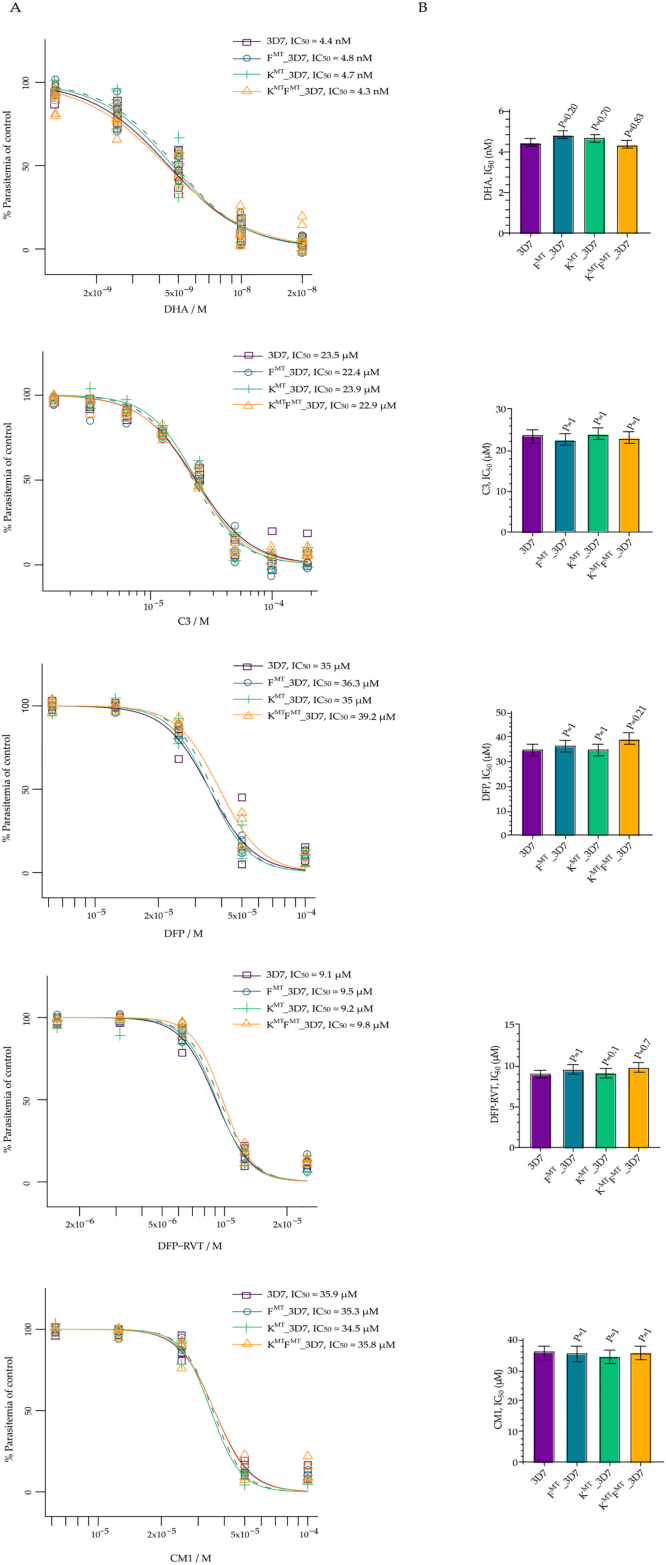
Dose-response analysis of growth inhibition for DHA, C3, DFP, DFP-RVT, and CM1. *P. falciparum* parental parasite 3D7 and transgenic parasites fd^D193Y^_3D7, k13^C580Y^_3D7, and k13^C580Y^fd^D193Y^_3D7 were treated with varying doses of each compound. (A) The dose-response graph plots for IC_50_ are shown. Data were obtained from at least three independent experiments and dose-response models were fitted to the data. The X axis indicates parasitemia as a term of percentage of non-treatment (100%), while the Y axis indicates compound concentration in molar units (M). The IC_50_ values acquired from the graph are annotated at the upper right corner. (B) The bar graphs represent a statistical comparison of IC_50_ values between the different parasite lines. The X axis indicates the IC_50_ value in micromolar units (µM). The IC_50_ values and the associated confidence intervals are shown. *P*-values from pair-wise t-tests with Bonferroni-Holm *post-hoc* correction comparing the IC_50_ of each transgenic parasite to that of the parental 3D7 parasite are shown above the IC_50_ values. A 0.05 *P*-value is used to indicate any difference. F^MT^ = fd^D193Y^ mutation, K^MT^ = k13^C580Y^ mutation.

The growth inhibitory properties of DHA, C3, DFP, DFP-RVT, and CM1 were assessed in *P. falciparum* parental strain 3D7 and the transgenic parasites fd^D193Y^_3D7, k13^C580Y^_3D7, and k13^C580Y^fd^D193Y^_3D7 (Kampoun et al., 2022). The potency of the compounds varied as follows: DHA > DFP-RVT > C3 > DFP ≈ CM1, in which the IC_50_ 95% confidence intervals for DHA, DFP-RVT, C3, DFP, and CM1 were 4–5 nM, 8–10 µM, 21–25 µM, 32–41 µM, and 32–38 µM, respectively. However, the IC_50_ values of the compounds were not significantly different between the 3D7 parental strain and the transgenic strains with *k13* and/or *fd* mutations (Fig. 2).

### Test of pharmacological interaction between DHA and test compounds in *P. falciparum*

**Figure 3:**
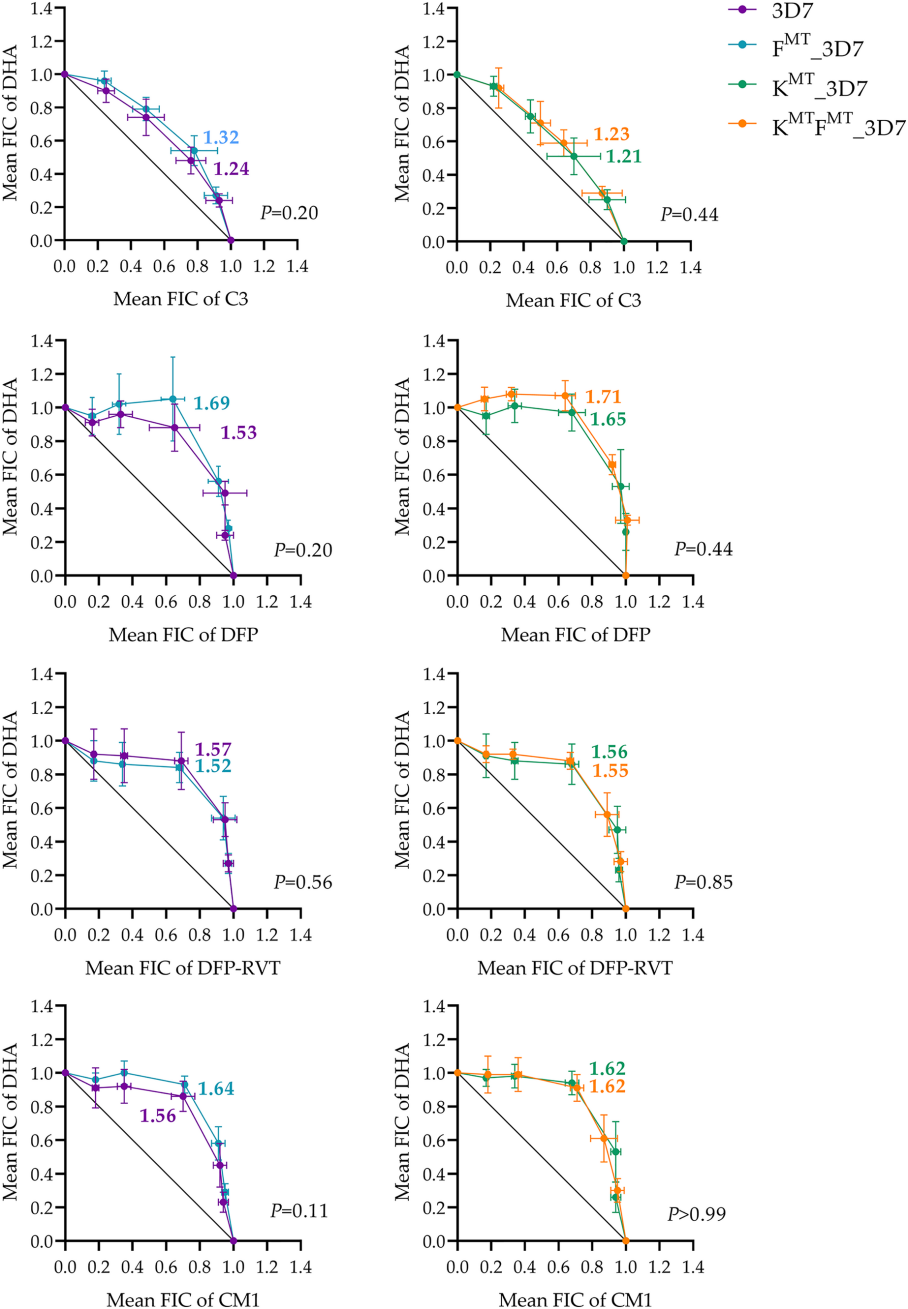
Isobologram testing the interaction between DHA and test compounds (candidate Fd-FNR inhibitor C3 or iron chelators DFP, DFP-RVT, and CM1) against parental 3D7 and transgenic F^MT^_3D7 (*fd* mutant, *k13* wild-type), K^MT^_3D7 (*fd* wild-type, *k13* mutant), and K^MT^F^MT^_3D7 (*fd* mutant, *k13* mutant) *P. falciparum*. The isobologram was generated by plotting the mean FIC values of DHA against the mean FIC values of C3 or the iron chelators. Data were obtained from at least three independent experiments; mean values are shown and error bars represent SD. The ΣFIC values for each combination are shown next to the isobologram. Statistical comparisons of ΣFIC values between *fd*-mutated and *fd* wild-type parasites were performed with a pairwise *t*-test, for which the *P* value is given at the lower right corner of each graph. F^MT^ = fd^D193Y^ mutation, K^MT^ = k13^C580Y^ mutation. ΣFIC < 0.9 indicates synergistic, 0.9 < ΣFIC < 1.1 indicates additive, and ΣFIC > 1.1 indicates antagonism.

The interactions between DHA and the test compounds were assessed as the fractional inhibition concentration (ΣFIC) index, and the mean ΣFICs of the combinations were compared between *fd* wild-type (fd^WT^) and *fd*-mutated (fd^MT^) parasites using Welch’s t-test (Fig. 3). An antagonistic effect was indicated when ΣFIC > 1.1, an additive effect was indicated when 0.9 < ΣFIC < 1.1, and synergism was indicated when ΣFIC < 0.9, using the criteria established by previous studies of antimalarial interactions (Chou, 2006; Crisafulli et al., 2021). For the DHA and C3 combinations, the mean ΣFICs were moderately antagonistic, which were 1.24, 1.32, 1.21, and 1.23 in the parental 3D7, fd^MT^_3D7, k13^MT^_3D7, and k13^MT^fd^MT^_3D7 mutants, respectively. Moreover, no significant differences in mean ΣFIC were observed between fd^WT^ and fd^MT^ parasites. All combinations of DHA with iron chelator compounds were antagonistic, although no significant difference was observed between the fd^WT^ and fd^MT^ parasites.

## Discussion

ART resistance is modulated by several factors, including the genetic background and pharmacological interactions with other antimalarials. For example, the *crt* and *mdr1* genes affect parasite response to Ca^2+^/Na^+^ channel blockers and their interactions with DHA (Eastman et al., 2016). Here, we investigated the interaction of C3, an inhibitor of the *Pf*Fd-*Pf*FNR protein interaction, with DHA in parasites with different *fd* and *k13* genetic backgrounds. C3 demonstrated moderate antagonistic activity toward DHA. Our hypothesis that inhibition of the Fd/FNR redox system in the apicoplast by C3 antagonizes DHA is in line with a recent report that other apicoplast-targeting antimalarials also antagonize DHA (Crisafulli et al., 2021). It should be noted that the degree of DHA antagonism by C3 cannot be directly compared with apicoplast targeting drugs reported previously (Crisafulli et al., 2021) owing to substantial methodological differences. Defects in the key apicoplast functions of isoprenoid metabolism and protein prenylation are thought to antagonize ART by the consequent reduction in hemoglobin trafficking (Crisafulli et al., 2021). Reduction in hemoglobin trafficking reduces the level of free heme from hemoglobin digestion, which is the main catalyst for ART activation. These findings caution against the use of ART partner drugs that inhibit Fd-FNR interaction and other apicoplast targets, which may reduce ART efficacy in malaria chemotherapy.

The antimalarial potency of the Fd-FNR inhibitor C3 was modest (IC_50_ 95% confidence interval 23–25 µM) and did not differ between 3D7 and genome-edited parasites, including those with the *fd* D193Y mutation. From the study of C3 docked with Fd, C3 was predicted to bind Fd at a site distant from the D193Y mutation (Suwito et al., 2014), which might explain why no difference in antimalarial potency was observed between the fd^WT^ and fd^MT^ parasites. In contrast, the antimalarial effect of C3 could be due to the inhibition of targets other than Fd, as the antimalarial IC_50_ is markedly lower than the concentration of C3 required for 50% inhibition of the interaction between recombinant Fd and FNR proteins (100 µM) (Suwito et al., 2014). The potential for promiscuous targeting by the chalcone derivative C3 is plausible because other chalcone derivatives are known to be promiscuous-targeting antimalarials, such as licochalcone A, the first identified anti-malarial chalcone derivative, which inhibits *P. falciparum* mitochondrial complexes II and III, and possibly the erythrocyte membrane (Mi-Ichi et al., 2005; Ziegler et al., 2004). Moreover, phenylurenyl chalcones, which are effective against quinoline-resistant *P. falciparum*, inhibit parasite cysteine proteases involved in hemoglobin degradation and hemozoin formation (Domínguez et al., 2005), and chalcone-chloroquine-based hybrids exert antimalarial activity by inhibiting hemozoin formation (Guantai et al., 2011; Sashidhara et al., 2012). In this study, the tested iron chelators showed modest antimalarial activity, similar to previous reports (Chuljerm et al., 2021; Ferrer et al., 2012; Maneekesorn et al., 2021; Thipubon et al., 2015). No differences in sensitivity to DHA were observed among the parasites tested, although it should be noted that the ART-resistance phenotype manifested in *k13* mutants is not detectable by the growth inhibition assay employed in this study (Ariey et al., 2014).

Importantly, the interactions of C3 with DHA in different parasite backgrounds were moderately antagonistic; hence, inhibition of the Fd-FNR interaction by C3 could antagonize DHA. Nevertheless, there was no significant difference between the fd^WT^ and fd^MT^ parasites, even in the parasite that contains both *fd* and *k13* mutations. Therefore, the antimalarial mode of action of C3 remains unclear. As expected, the iron chelators antagonized the antimalarial activity of DHA. As no significant differences in the mean ΣFICs between fd^WT^ and fd^MT^ parasites were observed, it can be concluded that this *fd* mutation does not affect the antagonistic interaction between DHA and iron chelators. Presumably, *fd* mutations associated with the loss of Fd function have a negligible impact on the iron pool targeted by iron chelators. The effect of the *fd* mutation on the interaction with DHA might be smaller than that of C3, such that it could not be detected by our experimental approach. Alternative approaches are required detect subtle interactions between DHA and apicoplast-targeting drugs. Apicoplast-targeting drugs typically exhibit a delayed-death antimalarial effect (Kennedy et al., 2019), such that interactions with the much faster-acting but short-lived DHA may not be detectable in assays with treatment conducted over one parasite replication cycle, as used in our study. To account for the different rates of killing, interaction assays can be performed by treating with apicoplast inhibitors for up to 72 h before pulsing with DHA (Crisafulli et al., 2021). Another possibility that should be considered is that the *fd*-D193Y mutation has no detectable effect on DHA interaction with other drugs, but is present at a high frequency among ART-resistant parasites because of positive selection to compensate for loss of fitness caused by *k13* mutation, or genetic hitchhiking with *k13* resistance mutations.

## Conclusions

The putative Fd-FNR inhibitor C3 demonstrated antimalarial activity comparable to that of iron chelators DFP, DFP-RVT, and CM1. No difference in sensitivity was observed among parasites with mutations in the *fd* and *k13* genes compared with the parental *P. falciparum 3D7* strain for any compound tested. In combination with DHA, C3 showed moderate antagonistic interactions, and iron chelators were antagonistic. Other approaches are required to understand the effect of the *fd* mutation on Fd function and how this affects the interaction of C3 with ART.

## Supplemental Information

10.7717/peerj.15187/supp-1 Supplemental Information 1 Raw Data for Figs. 1–3. (A) Fd int. IC_50_ values. (B) Fe chelators (IC_50_ values). (C) DHA (IC_50_ values). (D) DFP (IC_50_ values). (E) DFP-RVT (IC_50_ values). (F) CM1 (IC_50_ values).

## Additional Information and Declarations

### Data Availability

The following information was supplied regarding data availability: The raw data is available in the Supplemental File.
